# Effects of Combinatory In Vitro Treatment with Immune Checkpoint Inhibitors and Cytarabine on the Anti-Cancer Immune Microenvironment in De Novo AML Patients

**DOI:** 10.3390/cancers16020462

**Published:** 2024-01-22

**Authors:** Łukasz Bołkun, Aleksandra Starosz, Anna Krętowska-Grunwald, Tomasz Wasiluk, Alicja Walewska, Agnieszka Wierzbowska, Marcin Moniuszko, Kamil Grubczak

**Affiliations:** 1 Department of Haematology, Medical University of Bialystok, M. Sklodowskiej-Curie 24A, 15-276 Bialystok, Poland; 2 Department of Regenerative Medicine and Immune Regulation, Medical University of Bialystok, J. Waszyngtona 13, 15-269 Bialystok, Poland; aleksandra.starosz@umb.edu.pl (A.S.); anna.kretowska-grunwald@umb.edu.pl (A.K.-G.); alicja.walewska@umb.edu.pl (A.W.); marcin.moniuszko@umb.edu.pl (M.M.); 3 Department of Pediatric Oncology and Hematology, Medical University of Bialystok, J. Waszyngtona 17, 15-274 Bialystok, Poland; 4 Regional Centre for Transfusion Medicine, M. Sklodowskiej-Curie 23, 15-950 Bialystok, Poland; twasiluk@rckik.bialystok.pl; 5 Department of Hematology, Medical University of Lodz, Pabianicka 62, 93-513 Lodz, Poland; agnieszka.wierzbowska@umed.lodz.pl; 6 Department of Allergology and Internal Medicine, Medical University of Bialystok, M. Sklodowskiej-Curie 24A, 15-276 Bialystok, Poland

**Keywords:** acute myeloid leukaemia, cytarabine, immune checkpoint inhibitors, CTLA-4, PD-1, PD-L1

## Abstract

**Simple Summary:**

Immune checkpoint inhibitors’ (ICIs) therapeutic use remains a challenge in acute myeloid leukaemia (AML). We revealed the beneficial influence of the ICIs on the anti-cancer responses in the cancer in vitro chemotherapy setting. Anti-PD-1 or anti-PD-L1 antibodies had the most significant effect on the immune microenvironment of the AML. The blocking of the PD-1/PD-L1 axis induced the activation and proliferation of lymphocytes, with concomitant balance preservation through the modulation of immunosuppressive factors.

**Abstract:**

Despite substantial progress in the diagnostic and therapeutic procedures, acute myeloid leukaemia (AML) still constitutes a significant problem for patients suffering from its relapses. A comprehensive knowledge of the disease’s molecular background has led to the development of targeted therapies, including immune checkpoint inhibitors, and demonstrated beneficial effects on several types of cancer. Here, we aimed to assess in vitro the potential of the immune checkpoint blockage for supporting anti-cancer responses to the AML backbone therapy with cytarabine. PBMCs of AML patients were collected at admission and, following the therapy, eight complete remission (CR) and eight non-responders (NR) subjects were selected. We assessed the effects of the in vitro treatment of the cells with cytarabine and the immune checkpoint inhibitors: anti-CTLA-4, anti-PD-1, anti-PD-L1. The study protocol allowed us to evaluate the viability of the cancer and the immune cells, proliferation status, phenotype, and cytokine release. Anti-PD-L1 antibodies were found to exert the most beneficial effect on the activation of T cells, with a concomitant regulation of the immune balance through Treg induction. There was no direct influence on the blast cells; however, the modulation of the PD-1/PD-L1 axis supported the expansion of lymphocytes. Changes in the response between CR and NR patients might result from the differential expression of PD-1 and PD-L1, with lower levels in the latter group. The tested blockers appear to support the anti-cancer immune responses rather than directly improve the effects of cytarabine. In conclusion, checkpoint proteins’ modulators might improve the anti-cancer responses in the tumour environment.

## 1. Introduction

Acute myeloid leukaemia (AML) is a highly aggressive and heterogeneous haematologic malignancy, deriving from the myeloid stem cell [1,2]. A better understanding of the molecular background of the disease is crucial for further enhancement of the current AML treatment and for improving relapse-free survival with novel therapies [3]. Given their successful incorporation in the therapy of solid tumours, intensifying the anti-tumour immunity through immune checkpoint inhibitors has gained interest over the last few years [4].

Programmed cell death protein 1 (PD-1), an immune checkpoint expressed on activated T cells, NK cells, and B cells, is a crucial factor in promoting self-tolerance by downregulating immune responses [5]. Blocking the immune cell activation through the PD-1/PD-L1 axis has been identified as a potential pathway of immune evasion used by tumour cells [6]. According to Zhou et al., disease progression results in an increase in regulatory T cells (Tregs) and an elevated expression of PD-1 on CD8+ lymphocytes in a pre-clinical AML murine model [7]. Another checkpoint receptor—cytotoxic T-lymphocyte antigen-4 (CTLA-4)—is a surface molecule predominantly expressed on activated T cells and Tregs. The expansion of the latter population has already been observed in the course of AML [8]. Through a competitive bind to CD80 and CD86, CTLA-4 inhibits effector T cell activation, leading to the dampening of excessive immune reactions and maintaining homeostasis [9,10].

Numerous approaches have been explored in the context of immune checkpoint inhibitor implementation in AML [11]. The targeting of the CTLA-4 and PD-1/PDL-1 axis is being investigated in several clinical trials as a potential asset in the novel management of patients with AML. To date, in therapy-resistant or relapse post-allogenic stem cell transplantation settings, the response rate has been demonstrated to range from 22 to 72% [12,13,14]. This ambiguity may be caused by a higher heterogeneity and a lower mutational burden of AML compared to solid tumours [2]. There are also conflicting reports regarding the relationship between CTLA-4 ligands on the tumour cells—CD80/CD86 and the patients’ clinical outcome. Differences might result inter alia from the confirmed interaction of CD80/CD86 also with lymphocytes co-stimulatory CD28 molecule, which maintain cells responsiveness after TCR activation. Whiteway et al. showed that a higher co-expression of CD80/CD86 molecules on AML lymphoblasts leads to a longer disease-free survival [15]. On the contrary, Hock et al. reported that an elevated concentration of the soluble CD86 (sCD86) in adult patients was associated with a worse prognosis [16]. A blockage of immune checkpoints is predominantly aimed at supporting the immune response; therefore, combinations with the currently used chemotherapeutics, i.e., azacytidine or cytarabine, are of great importance [11].

Implementation of cytarabine (Ara-C) is one of the most common approaches in AML treatment. Despite the toxicity-related side effects, we observe an increased percentage of a relapse-free survival [17,18]. Nevertheless, considering the life-threatening toxic effect, high doses are not recommended for older patients. Hypomethylating agents, such as azacitidine, have for years been demonstrating promising effects in AML management. According to Daver et al., the antitumorigenic role of azacitidine may be concomitantly decreased by its impact on the immune checkpoint upregulation, namely CTLA-4, PD-1, and PD-L1, causing a reduction in the immune responses [19]. Progressing resistance to chemotherapy results in a decline in the AML patients’ survival rate. That constitutes a foundation for novel therapeutic agents’ research that could enhance the effectiveness of cytarabine [20].

Despite recent advances in genomic profiling and the introduction of targeted therapy, the prognosis of patients with a relapsed or refractory AML remains poor. Therefore, cytarabine is still the backbone of the leukaemia management [21]. A concomitant or consecutive administration of immune checkpoint inhibitors and chemotherapy can be encouraging, although studies on the subject are still very scarce [4,12,22,23]. Regarding the nature of AML and its interactions within the tumour microenvironment, a combination of immunotherapy and standard chemotherapeutics seems to be a promising direction for treatment [24,25]. In this study, we focused on the in vitro evaluation of the immune checkpoint blockage effects on both the modulation of the anti-cancer immune responses and support for the chemotherapeutic action against AML blasts. We implemented a retrospective clinical stratification of patients into those responding and not responding to the therapy and assessed differences in the inhibition application efficiency in both groups.

## 2. Materials and Methods

### 2.1. Patients’ Description

The samples were collected upon obtaining written informed consent pursuant to the rules and tenets of the recently revised Helsinki protocol. Patients’ median age was 59.5 years (in the range of 45.5 to 61.75 years). Eight subjects were females and eight males. Blood counts and flow cytometry were used to confirm the presence of blastic cells. Cytogenetic and molecular studies were performed in accordance with the European LeukemiaNet (ELN) 2017 recommendation [26]. All patients involved had a normal karyotype (46XX/46XY), and none of them suffered from the mutated core binding factor leukaemia (CEBPA*_mut_*), mutated nucleophosmin (NPM1*_mut_*), or internal tandem duplication of Fms-like tyrosine kinase 3 (FLT3-ITD). AML patients were treated with seven-day induction chemotherapy regimens corresponding to the standard therapy based on the Polish Adult Leukaemia Group (DAC schedule) [27]. After, the morphological response was evaluated; eight patients achieved complete remission (CR) after the 1st induction and eight were non-responders (NR). Peripheral blood mononuclear cells (PBMC) were isolated from blood using density gradient—Pancoll 1.077 g/L (PAN Biotech, Aidenbach, Germany). Following centrifugation, buffy coat containing mononuclear cells was obtained (lymphocytes and monocytes). Following additional washing steps with phosphatate-buffered saline (PBS; Corning, Corning, NY, USA), PBMCs were stored at −196 °C (liquid nitrogen; long-term storage) in cryoprotectant: 10% DMSO (Sigma-Aldrich, Burlington, MA, USA) in fetal bovine serum (FBS; PAN Biotech, Aidenbach, Germany). Trypan blue was used for assessing viability of the samples thawed for the experiments (viable cells frequency around 94–98%). Characteristics of the selected patients are provided within the supplementary materials (Figure S1A). The informed consent was obtained from all the subjects. The experimental protocol was approved by Local Bioethical Committee in Bialystok, approval number: R-I-002/393/2018. 

### 2.2. AML Patients PBMC Culture with Immune Checkpoint Inhibitors and Cytarabine Presence

Isolated PBMCs of 16 patients with AML were used for testing of immune checkpoint inhibitors with/without cytarabine (Alexan, Ebewe Pharma, Unteracht, Austria). Group included samples from 8 CR and 8 NR patients (Figure S1A). The PBMC was suspended in DMEM medium (PAN Biotech) enriched with fetal bovine serum (FBS, PAN Biotech) and antibiotic (gentamicin; Gibco, Grand Island, NY, USA) at 2 × 10^6^ cells/mL. Cells for proliferation assessment were stained with CFSE (Sigma-Aldrich). AML blasts were incubated in the presence of a chemotherapeutic drug—cytarabine (Alexan)—at the concentration of 40 μM. Cytarabine concentration was based on its calculated approximate concentration in the blood after the IV administration of the 100 mg/m^2^ of Alexan at the induction phase into around 5.5 L of blood (to include pharmacokinetics effects in organism, the dose used in vitro was reduced by half). In addition, dose of the cytarabine used affected blast cells but preserved population of lymphocytes (Figure S2A). Within these arrangements, the following immune checkpoint inhibitors were tested: anti-CTLA-4 (clone AS32, monoclonal IgG1), anti-PD-1 (polyclonal IgG), anti-PD-L1 (monoclonal IgG), each at concentration of 1 μg/mL (based on the previous experience in breast cancer cells [28], where demonstrated doses showed effective modulation of the immune response). Importantly, to confirm validity of the blocking factors used, we performed initial screen of tested immune checkpoint proteins on the surface of blasts and lymphocytes (Figure S2B). Controls with no blocking proteins and only with the presence/absence of cytarabine were included. Non-proliferating cells, as a reference for the expansion analysis using CFSE, were inhibited by 25 ng/mL of colcemide (Demecolcin; Biowest, Nuaille, France). Cells were cultured in 5% CO_2_, 37 °C, for 48 h (or 96 h for the proliferation test). Implemented incubation conditions allowed for the maintenance of high viability and proliferative status of both blast cells and lymphocytes within monitored time period without any additional stimulation (Figure S2C,D).

### 2.3. Flow Cytometric Evaluation of Changes Induced In Vitro by Immune Checkpoint Blockers, with/without Cytarabine in AML Blasts

Following incubation, cells were collected and washed with PBS and stained with fluorochrome-conjugated monoclonal antibodies. AML patients’ cells were stained with anti-CD13 FITC (clone SJ1D1) and anti-CD33 FITC (clone P67.6) (BD Bioscience; San Jose, CA, USA). Unstained and FMO (fluorescence minus one) controls were used to establish a proper gating strategy. The data were acquired on FACS Calibur flow cytometer (BD Bioscience; San Jose, CA, USA) and processed with FlowJo software 9.5.1 (TreeStar Inc., Ashland, OR, USA). Analysis of the viability within the cells of interest was performed using 7-aminoactinomycin D dye (7AAD, BD Bioscience). Blasts were gated on the basis of CD13/CD33 and morphological properties (FSC, forward scatter—relative size; SSC, side scatter—relative internal structure). A percentage of dead cells (7AAD-positive) was assessed in blasts and lymphocytes (Figure S1B). The proliferation was analysed within CD13/CD33+ blast cells and lymphocytes on the basis of CFSE expression and gating excluding non-proliferating cells with colcemide control. Blasts were stained with anti-CD13 PE (clone L138) and anti-CD33 (clone D3HL60.251) (Beckman Coulter, Brea, CA, USA). 7AAD dye was used to exclude dead cells from the analysis. The proliferation status was established in AML blasts and lymphocytes in accordance with the gating strategy (Figure S1C).

### 2.4. Cytometric Analysis of AML Lymphocytes Response to In Vitro Culture with Immune Checkpoint Inhibitors with/without Cytarabine

Fluorochrome-conjugated monoclonal antibodies were used for PBMC staining, including anti-CD4 FITC (clone RPA-T4), anti-CD8 PE (clone HIT8α), anti-CD25 PE-Cy5 (clone M-A251), anti-CD127 AlexaFluor647 (clone HIL-7R-M21) (BD Bioscience). Stained samples were run on flow cytometer and analysed using FlowJo software. Initially, lymphocytes were distinguished on the basis of their FSC and SSC properties. Next, populations of CD4+ (Th, helper T cells) and CD8+ (Tc, cytotoxic T cells) lymphocytes were delineated. The expression of CD25 (IL-2Ra; activation marker) and CD127 (IL-7R; development-related marker) were assessed within the gated subsets of lymphocytes (Figure S1D). In addition, regulatory T cells (Tregs) were gated and analysed in the context of the frequency within the lymphocytes. The gating strategy for Tregs was based on the presence of a high CD25 expression and low/no CD127 on T cells’ surface (Figure S1E). Unstained and FMO controls were used for setting gating strategies (Figure S3A–D).

### 2.5. Evaluation of Cytokines Released by AML PBMC Incubated with Immune Checkpoint Inhibitors with/without Cytarabine

Supernatants were used to analyse the release of cytokines. Immunoenzymatic DuoSet ELISA Kits (R&D system, Minneapolis, MN, USA) were used in accordance with the protocols provided by the manufacturer. Evaluated cytokines were related to pro- and anti-inflammatory reactions: IL-1beta, IL-6, IFN-gamma, IL-10, TGF-beta, IL-17, TNF-alpha. The absorbance associated with the presence of cytokines was acquired at 450 nm wavelength using LEDETECT96 microplate reader with MicroWin2013 software (Labexim Products, Lengau, Austria). The final concentrations were calculated on the basis of the standard curve (four-parameter logistic (4-PL) curve-fit).

### 2.6. RT-PCR Analysis of Immune Checkpoint Proteins and Immune Cells-Related Transcription Factors in PBMC of AML Patients

Isolation of mRNA from the PBMC was performed using RNeasy Micro Kits (Qiagen, Hilden, Germany) according to the manufacture protocol. The amount of mRNA was quantified on NanoDrop (Thermo Fisher Scientific; Waltham, MA, USA). The calculation was based on the evaluation of the A260/A280 absorbance ratio. The mRNA of each patient was subjected to the reverse transcription using the High-Capacity cDNA Reverse-Transcription Kit (Applied Biosystems; Foster City, CA, USA), with the standard incubation protocol: primers annealing (10 min, 25 °C), DNA polymerization (120 min, 37 °C), reverse transcriptase deactivation (5 min, 85 °C). Real-time qPCR was carried out using the TaqMan Fast Advanced Master Mix and pair of primers with TaqMan FAM-labeled probes (Applied Biosystems; Foster City, CA, USA). Tested genes included *CTLA-4* (Hs00175480_m1), *PDCD1* (Hs01550088_m1), *PDL1* (Hs00204257_m1), *FOXP3* (Hs01085834_m1), *RORC* (Hs01076112_m1), *GATA3* (Hs00231122_m1)*, TBX21* (Hs00894392_m1), and *STAT6* (Hs00598625_m1), together with two housekeeping genes—*GUSB* (Hs99999908_m1) and *HPRT1* (Hs99999909_m1). Following reaction stages were applied: 1 cycle of activation of enzyme (95 °C, 20 s) and 40 cycles of denaturation (95 °C, 1 s) and annealing with elongation (60 °C, 20 s), respectively. StepOnePlus Real-Time PCR System device (Applied Biosystems; Foster City, CA, USA) and StepOnePlus software v2.3 was used for RT-PCR analysis. Data were presented as a relative expression (log_2_(2^−dCt^)) normalised versus *GUSB* and *HPRT1*.

### 2.7. Statistical Analysis

Biostatistical analysis of the data was performed with GraphPad Prism 8.0 software (GraphPad Software Inc., San Diego, CA, USA). Two-way ANOVA (full model fitted and Geisser–Greenhouse correction) and Fisher LSD tests were implemented for the unified and strictly standardized in vitro conditions. Results are presented as a percentage mean change in reference to the cells treated only with cytarabine (set as 100%). Statistically significant/essential differences were indicated with red/blue asterisks/*p*-values when comparing the effects of blockers versus cytarabine only. Additionally, variations in the response between CR and NR patients were demonstrated with black brackets and asterisks. In order to evaluate the associations between the tested parameters, the Pearson correlation analysis was applied to establish coefficient values. The results were presented with heatmaps demonstrating r values (with red and green colour for positive and negative correlations, respectively), together with an indication of their statistical significance using asterisks or an exact p value. Statistical significance threshold was set to *p* = 0.05, and the subsequent levels of significance included: *p* < 0.05—*, *p* < 0.01—**, *p* < 0.001—***, *p* < 0.0001—****.

## 3. Results

### 3.1. Immune-Related Changes Occurring in the Course of In Vitro Treatment of AML PBMC with Immune Checkpoint Inhibitors in the Presence of Cytarabine

**Figure 1:**
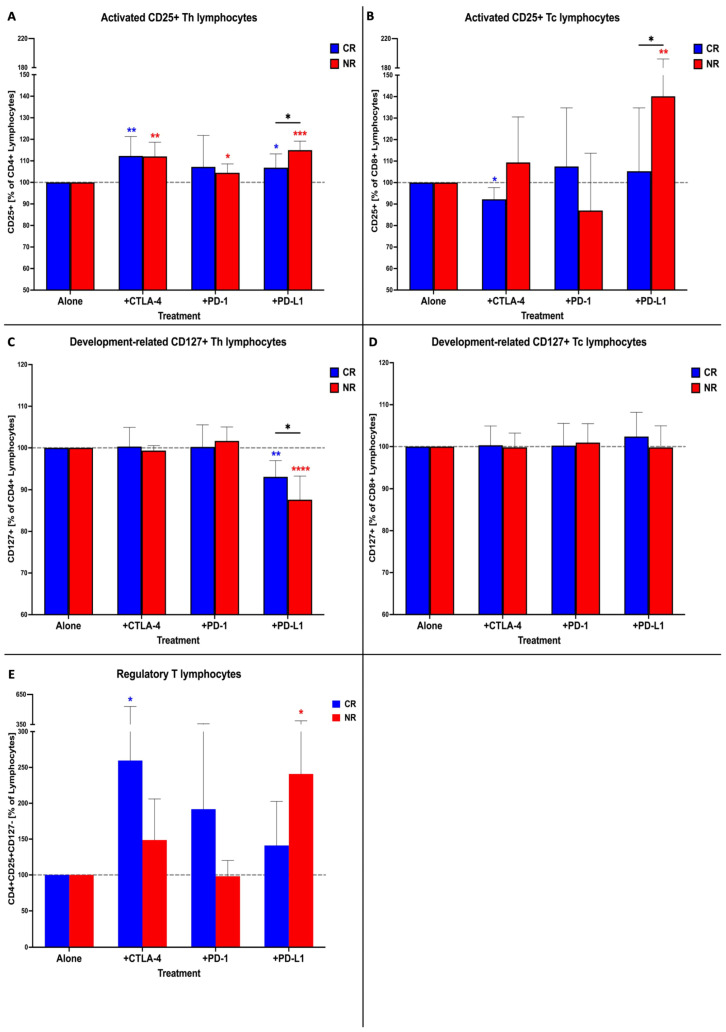
Assessment of lymphocyte-related parameters in AML PBMC treated in vitro with immune checkpoint inhibitors and cytarabine. Influence of immune checkpoint inhibitors’ effect on activation status (CD25 expression) of CD4+ (**A**) and CD8+ (**B**) lymphocytes. Effects of tested inhibitors on development status (CD127 expression) of CD4+ (**C**) and CD8+ (**D**) lymphocytes. Verification of immune checkpoint blockage effects on frequency of Tregs (**E**). Statistical significance indicated with *p* value or asterisks: *p* < 0.05—*, *p* < 0.01—**, *p* < 0.001—***, *p* < 0.0001—****.

Experiments involving immune checkpoint inhibitors demonstrated diverse responses of AML patients’ lymphocytes. We revealed an increase in the number of activated CD25+ Th cells in both the CR and NR subgroups, especially when treated with the anti-CTLA-4 or anti-PD-L1 antibodies. The NR patients’ CD4+ Th cells responded in a stronger manner to anti-PD-L1 compared to the CR group (Figure 1A). In CD8+ Tc cells, the changes were demonstrated when blocking PD-L1 in the NR group. A slight reduction in the activation of CD8 lymphocytes was observed in the CR group treated with anti-CTLA-4 (Figure 1B). We did not find immune checkpoint inhibitors to have a critical impact on the frequencies of CD127+ cells (Figure 1D). An exception was observed in CD4+ Th cells, where the anti-PD-L1 antibodies reduced the frequency of CD4+ Th cells with the development-related marker (CD127) in both the CR and NR subgroups (Figure 1C). Blocking with anti-CTLA-4 was found to increase the frequency of regulatory T cells (Tregs) in the CR group. Higher levels of Tregs were observed in the NR group in response to the anti-PD-L1 antibodies (Figure 1E).

**Figure 2:**
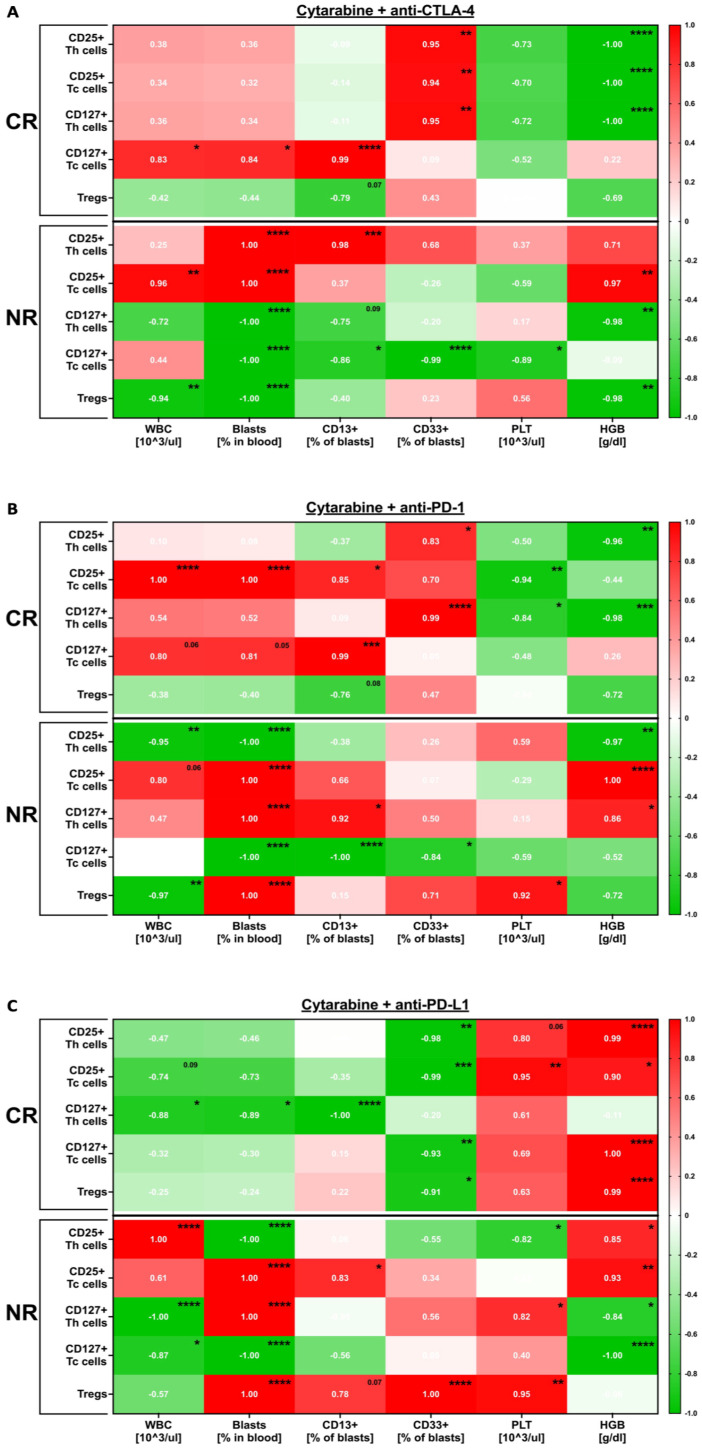
Evaluation of relation between AML patients’ haematological data and immune cells responses in vitro to therapeutics. Correlation analysis of associations between laboratory tests-based haematological status of the AML patients and their response to immune checkpoint inhibitors in vitro in the setting, including anti-CTLA-4 (**A**), anti-PD-1 (**B**), and anti-PD-L1 (**C**) antibodies. Statistical significance indicated with *p* value or asterisks: *p* < 0.05—*, *p* < 0.01—**, *p* < 0.001—***, *p* < 0.0001—****.

The assessment of patients’ haematological results together with Immunological in vitro data revealed significant correlations. Changes in CD25+ lymphocytes treated with anti-CTLA-4 were shown to be related to CD33-positive blasts in the blood: positively in the CR and negatively in the NR group. Inversed dependencies were observed in the context of the level of haemoglobin. The haematological data of our CR and NR patients demonstrated negative correlations with the levels of CD127+ T cells. These parameters demonstrated a positive correlation in only the CR group. Tregs, leukocyte, and blast blood levels seemed to be associated with the reduced frequencies of those T cells in our NR patients (Figure 2A). Regarding the inhibition of PD-1, the most significant difference was the negative correlation of leukocytes and blasts with activation and the development status of Th and Tc cells, respectively, in the NR group. Blasts and white blood cells correlated positively with the activation of Tc lymphocytes; however, NR patients demonstrated a negative relation between these parameters and the development of that T cell subpopulation. Similarly to anti-CTLA-4 data, here, only the NR group showed a correlation of haematological data with Tregs (Figure 2B). Fluctuations in those cells in PD-L1 inhibition were strongly related to the clinical data. Responses to anti-PD-L1 antibodies were found to be more dependent on haematological parameters in the NR than CR patients. Those were especially associated with positive correlations with the activation and development status of the Tc lymphocytes (Figure 2C).

### 3.2. Effects Exerted In Vitro by Immune Checkpoint Inhibitors on AML Patients’ Blasts and Lymphocytes in the Presence of Cytarabine

**Figure 3:**
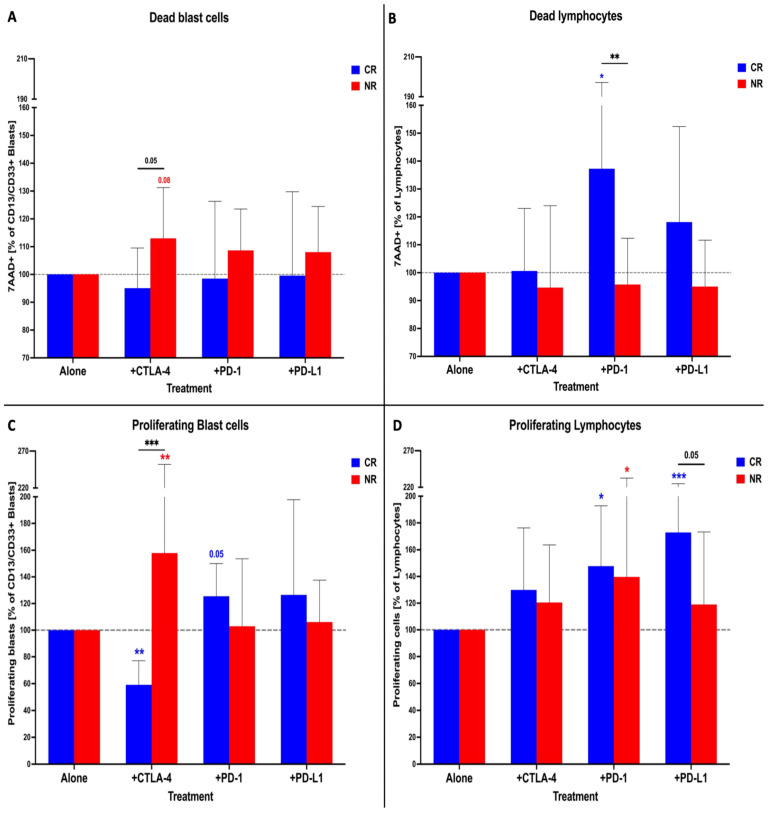
Immune checkpoint inhibitors in vitro effects on AML blasts and lymphocytes. Viability changes in CR or NR patients’ groups within blasts (**A**) and lymphocytes (**B**). Proliferative responses to blocking of the immune checkpoint proteins in blast (**C**) and lymphocyte (**D**) population. Statistical significance indicated with *p* value or asterisks: *p* < 0.05—*, *p* < 0.01—**, *p* < 0.001—***.

The NR group treated with anti-CTLA-4 showed a tendency for an elevated level of dead blasts was reported. These were significantly higher compared to the CR patients (Figure 3A). Inhibitors of immune checkpoint proteins did not have an essential effect on the viability of lymphocytes, except for anti-PD-1 antibodies, which caused an increase in the frequency of dead cells in the CR group (Figure 3B). In the context of blast proliferation, the application of anti-CTLA-4 antibodies led to higher levels of that parameter in NR patients. On the contrary, the same setting caused a decline in blast proliferation in the CR group (Figure 3C). Interestingly, blocking CTLA-4 did not influence the expansion of lymphocytes, which responded only to the use of anti-PD-1 and anti-PD-L1 antibodies. Both our CR and NR subjects demonstrated higher proliferation in the presence of the PD-1 blocking factor. However, anti-PD-L1 led to an increased lymphocyte expansion in only the CR patients (Figure 3D).

**Figure 4:**
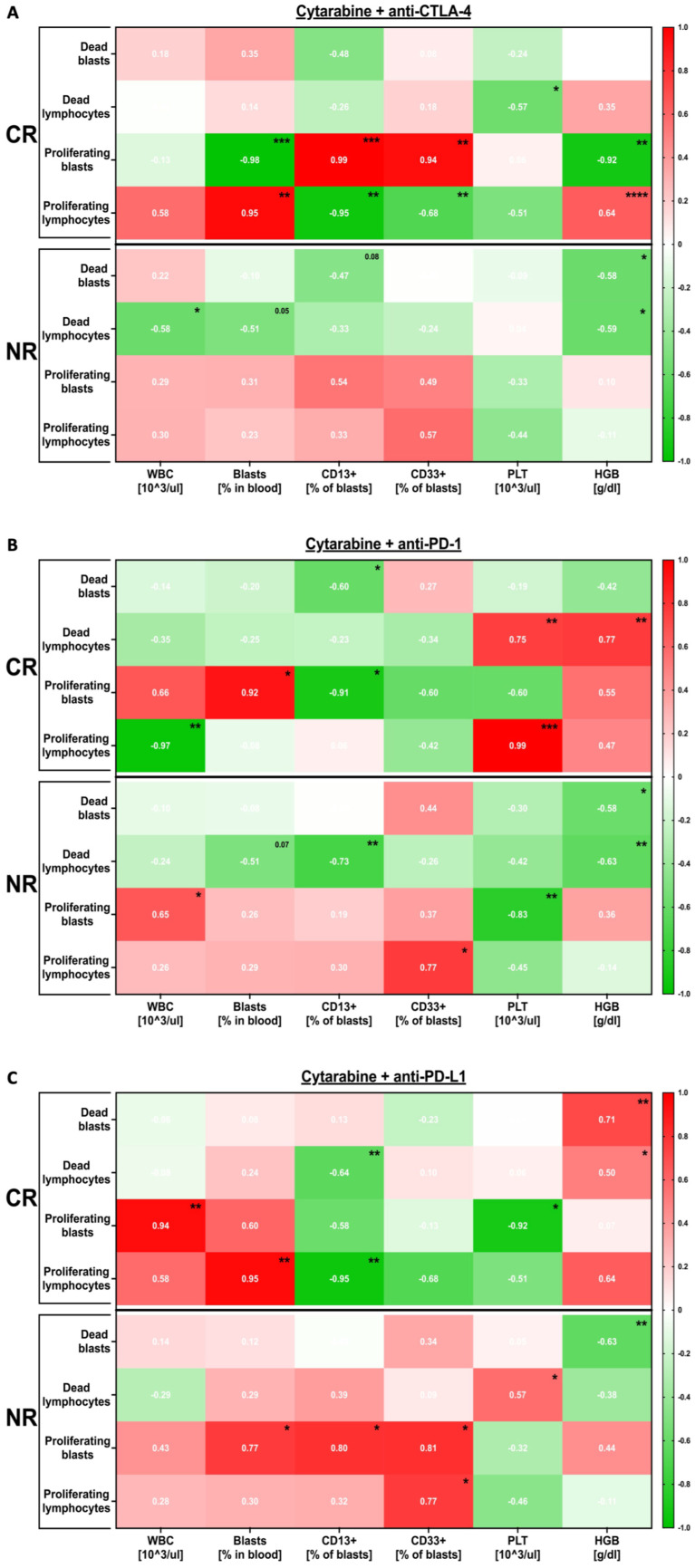
Correlation between AML patients’ haematological data and blasts’ responses to in vitro treatment. Relations between haematological data of the AML patients and their in vitro responses in the setting, including anti-CTLA-4 (**A**), anti-PD-1 (**B**), and anti-PD-L1 (**C**) antibodies. Statistical significance indicated with *p* value or asterisks: *p* < 0.05—*, *p* < 0.01—**, *p* < 0.001—***, *p* < 0.0001—****.

The haematological data seemed to be only essentially related to in vitro responses in the blasts and lymphocytes of CR patients in the setting with anti-CTLA-4 antibodies. We found that the frequencies of CD13+ and CD33+ blasts correlated positively and negatively with the blast and lymphocyte proliferation, respectively. Inversed relations were demonstrated when total blood blasts were analysed and were similar to the data concerning HGB (Figure 4A). Blasts’ proliferative responses to anti-PD-1 were found to demonstrate an opposite correlation with the total and CD13-positive blasts, predominantly in CR patients. Regarding the CR group, positive associations with blast or lymphocyte expansion were shown when linked to blood leukocytes or CD33+ blasts. Strong correlations were also reported between platelets (PLT) and anti-PD-1-induced changes in lymphocyte proliferation in the CR patients. Interestingly, in the NR group, PLT and HGB showed a negative association with proliferating and dead blast levels, respectively (Figure 4B). In the presence of anti-PD-L1 antibodies, the leukocytes and blasts in blood correlated strongly with the proliferative response of the blasts and lymphocytes in vitro. We found that HGB seems to be positively linked with the levels of dead blasts in the same setting of CR subjects, with opposite dependencies in the NR group (Figure 4C).

### 3.3. Evaluation of Selected Immune Checkpoint Proteins in AML Patients and Profiling of Immune Cells Phenotype-Related Markers

**Figure 5:**
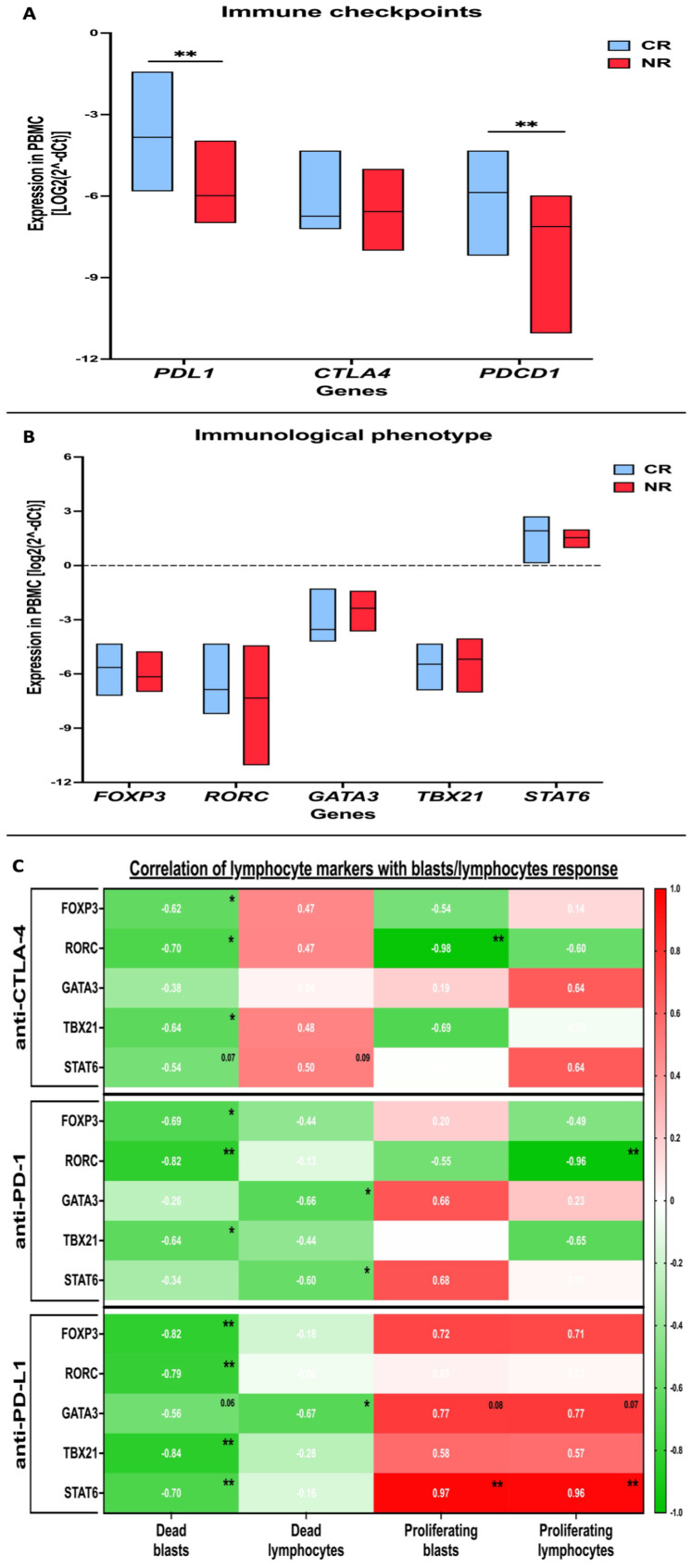
Profiling of immune checkpoint proteins and lymphocyte phenotype in AML patients PBMC. Gene expression of selected immune checkpoint proteins in CR and NR groups of AML patients (**A**). Assessment of transcription factors related to specific T cell subtypes: *Foxp3* (Treg), *RORC* (Th17), *TBX21* (Th1), *GATA3*, and *STAT6* (Th2) in AML subgroups (**B**). Correlation analysis between blast/lymphocyte response to in vitro therapy and lymphocyte phenotype-related markers (**C**). Statistical significance indicated with *p* value or asterisks: *p* < 0.05—*, *p* < 0.01—**.

Considering the reported differences in responses between our CR and NR patients, we aimed at verifying the expression levels of selected immune checkpoint proteins in AML patients’ PBMC. First, we verified the expression level of tested inhibitor targets on mRNA levels in both CR and NR AML patients’ blasts. No differences were reported in reference to the level of *CTLA4*. Furthermore, our CR patients demonstrated a significantly higher expression of the *PDL1* and *PDCD1* expression level compared to the NR group (Figure 5A).

With regard to the immune profiling, we verified whether our groups of CR and NR patients demonstrated differences in transcriptional factors closely associated with specific lymphocyte subsets: *Foxp3* (Treg), *RORC* (Th17), *TBX21* (Th1), *GATA3*, and *STAT6* (Th2). The performed analyses did not reveal significant differences between AML subgroups (Figure 5B).

The evaluation of associations between the immune cells and in vitro responses to CTLA-4 inhibition demonstrated a negative correlation of *Foxp3*, *RORC*, and *TBX21* with the frequency of dead blasts. Additionally, Th17 cells (*RORC*) were linked in the same manner with blast proliferation. A similar relation of T cell phenotypes to the frequency of dead blasts was shown in the setting with anti-PD-1 antibodies. However, *RORC* gene correlated negatively with lymphocyte proliferation. In addition, higher Th1- and Th2-related transcriptional factors were shown to be associated with a reduced frequency of dead lymphocytes. Dead blasts in the layout with anti-PD-L1 inhibitors were found to correlate negatively with all the tested transcriptional factors. The expression of *STAT6* and *GATA3* (Th2) positively correlated also with blast and lymphocyte proliferation (Figure 5C).

### 3.4. Cytokine Profile and Its Association with AML Patients’ PBMC Response to In Vitro Treatment with Immune Checkpoint Protein Inhibitors in the Presence of Cytarabine

**Figure 6:**
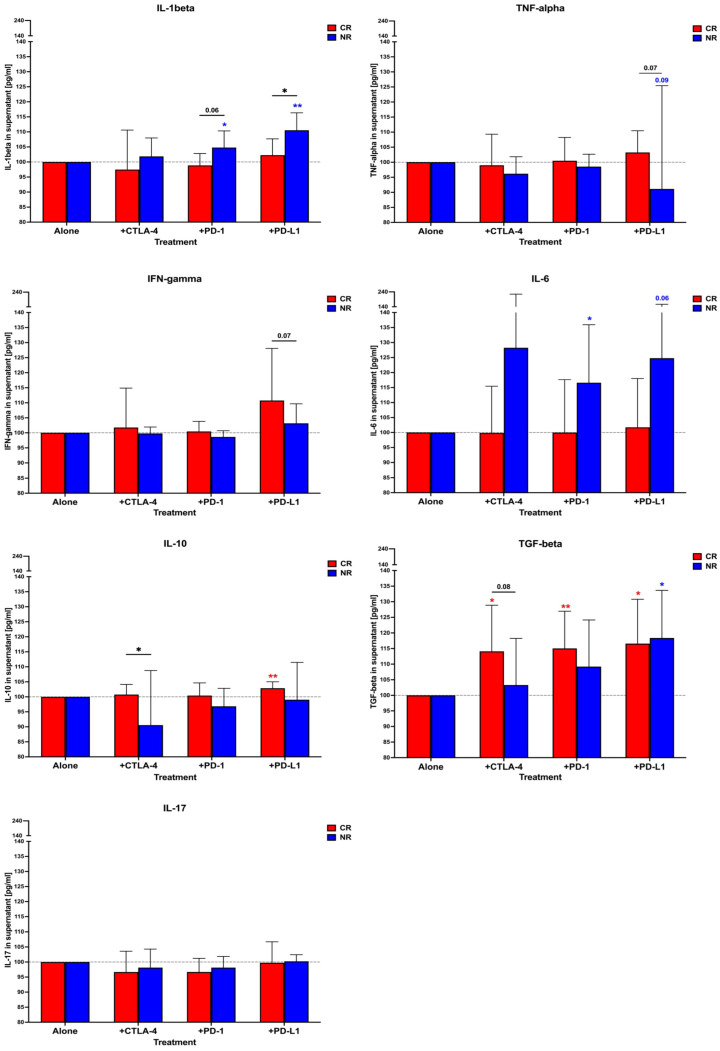
Profiling of cytokines released by AML patients PBMC in presence of immune checkpoint inhibitors. Changes in selected cytokines in CR and NR groups treated in vitro with cytarabine and immune checkpoint inhibitors: IL-1beta, TNF-alpha, IFN-gamma, IL-6, IL-10, TGF-beta, IL-17. Statistical significance indicated with *p* value or asterisks: *p* < 0.05—*, *p* < 0.01—**.

Release of pro-inflammatory cytokines in vitro by AML PBMC treated with anti-CTLA-4 antibodies revealed an increase in only the IL-6 level in the NR group; however, there was no statistical significance. Anti-inflammatory TGF-beta was elevated in CR patients compared to cytarabine alone but also in reference to the NR group. Reduced values, when comparing the NR to CR groups, were also reported in IL-10 production. In the samples with PD-1 blocking, we observed an increase in the release of IL-1beta and IL-6 by the NR patients’ cells. On the contrary, CR patients responded with elevated concentrations of TGF-beta. Comparable data were shown in the setting with anti-PD-L1 antibodies where the CR group demonstrated higher levels of both IL-10 and TGF-beta. PBMC of NR patients also produced more TGF-beta in the presence of PD-L1 inhibition. Importantly, these cells also released higher amounts of IL-1beta and IL-6. Noteworthy, IFN-gamma, TNF-alpha, and IL-17 did not play a significant role in the tested layouts and implemented immune checkpoint inhibitors (Figure 6 and Figure S4).

Subsequently, we aimed to assess associations between significantly changed cytokines and blast/lymphocyte responses to immune checkpoint inhibitors. In the anti-CTLA-4 antibody setting, IL-10 correlated negatively with the level of dead lymphocytes in the NR group. In CR patients, the presence of higher TGF-beta concentrations might be associated with reduced proliferation of lymphocytes but, concomitantly, with a higher rate of blast viability reduction. When blocking PD-1, the blasts of the NR group demonstrated a negative association with IL-1beta and IL-6. However, the same conditions demonstrated a link between a higher IL-1beta and reduced proliferation of lymphocytes. In the NR group, TGF-beta correlated positively with dead blast levels; however, these patients showed lower cytokine values compared to the CR group. PBMCs of CR patients treated with anti-PD-L1 antibodies demonstrated a positive correlation of higher concentrations of TGF-beta with increased frequencies of dead blasts. Simultaneously, the cytokine was negatively associated with the proliferation of lymphocytes. In the NR group, IL-6 was found to correlate positively with lymphocyte proliferation and blast proliferation. As the same patients demonstrated a tendency for reduced TNF-alpha levels, these changes could be associated with a lower frequency of dead blasts and inhibited lymphocyte proliferation (Figure S5A–C).

## 4. Discussion

Remarkable attention has been paid in recent years to the properties of immune checkpoint proteins in the context of their clinical implementation. The application of ipilimumab (anti-CTLA-4) in a combinatory therapy for stage III/IV melanoma patients was a historical step towards their introduction in cancer therapy [29]. It is believed that the blockage of these molecules combined with chemotherapy at a specifically designed dosage might exert beneficial effects, yet more research and clinical trials are still required [11,22,30].

The development of immune checkpoint therapies has provided a new therapeutic strategy for the treatment of haematology malignancies [31]. Recent studies have found that PD-1/PD-L1 are upregulated in myeloid malignancies, including AML. In addition, both cell culture and animal experiments have strongly suggested the potential benefits of PD-1/PD-L1 blockers in preventing the progression of this disease and potent clinical efficacy [32,33]. Currently, there are several clinical trials in de novo and refractory AML with various checkpoint inhibitors. In addition, the following were also tested with a combination of traditional chemotherapy inter alia: idarubicin and/or cytarabine and pembrolizumab/nivolumab (anti-PD-1) [22,34].

Recent trials have shown beneficial effects of the HiDAC (high-dose cytarabine) chemotherapy combined with pembrolizumab (anti-PD-1) in the refractory/relapsed AML [23]. On the contrary, we demonstrated that anti-PD-L1 antibodies in vitro seemed more efficient in increasing the activation and proliferation status of T cells. Noteworthy, we focused on reflecting the standard doses of cytarabine in the cell culture and used newly diagnosed patients with acute leukaemia. In the tested setting, more promising responses to PD-L1 might be associated with its comparably higher expression in tested PBMC. Beneficial effects were predominantly associated with the immune response restoration/improvement rather than a direct influence on the blast cells, which is in line with the trial mentioned above [23]. Regarding the use of PD-1 or CTLA-4 inhibition, comparable effects had already been presented, yet they regarded exclusively a generally improved response of T cells against AML blasts [35]. In the present study, we have further demonstrated that these effects were especially related to the activity and development of helper T cells. Although no crucial differences were shown between CR and NR patients treated with cytarabine and anti-PD-1, the implemented blockage was effective in inducing lymphocyte proliferation [22].

We showed here that, in the presence of the anti-cancer application of anti-PD-L1 antibodies, we can obtain an increase in the frequency of immunosuppressive Treg cells in NR AML patients. On the contrary, another study reported reduced Treg levels in the presence of PD-1, signaling blockage with the use of anti-PD-L1, together with a better survival of the mice with the lower Treg levels. However, those data corresponded to the responses of the HEL AML cell line with no stratification into CR and NR patients [36]. In addition, we involved an anti-cancer drug, cytarabine, in our model with human AML patients’ samples. Therefore, despite reduced Treg levels’ association with a better survival, as reported in the C1498 AML mice model [36], anti-PD-L1 use in AML management should be undertaken with caution considering other anti-cancer drugs implemented in the therapy. Here, in addition to assessing the responses of Tregs and activated T cells to an in vitro treatment, we also revealed their association with a clinical response to chemotherapy—with CR and NR stratification.

It was previously suggested that the expression of PD-L1 induces the Treg phenotype expansion, contributing to AML progression [36]. Our study revealed that PD-L1 is expressed differently between CR and NR patients. This could possibly explain the opposite responses of regulatory T cells between the tested groups when inhibiting PD-L1. We presume that higher PD-1 and PD-L1 in PBMC of CR patients might be associated with a better induction of lymphocyte activation in the PD-1/PD-L1-dependent pathway. Due to an increased presence of PD-1 on the surface of immune cells, anti-cancer responses could hypothetically be maintained even when partially locked by PD-L1 on cancer cells. On the contrary, the limited expression of PD-1 in the NR group, especially when inhibited with its ligand from blast cells, might completely deprive lymphocytes of physiological PD-1/PD-L1 axis activity. Analysing the expression of *CTLA4* in the PBMC of AML patients, we did not find differences between CR and NR subjects that could allow us to use this marker as a prognostic factor for the response to therapy. A study by Radwan et al., however, suggested that high levels of CTLA-4 are a good diagnostic marker of a poor prognosis in the AML (without CR/NR stratification) [37]. Gene profiling of the immune signatures in other studies suggested that high levels of CTLA-4, PD-1, or PD-L1 were associated with a worse therapy outcome [34,38]. However, we found that those who responded efficiently to the chemotherapy demonstrated a high expression of those genes at the pre-treatment stage. Our data could also be explained by the previously demonstrated better reduction of the expansion of blasts derived from AML subjects with a higher expression of PD-L1 (not demonstrated in the case of CTLA-4) [35]. Therefore, risk evaluations described above might not always correspond to the clinical outcome, as our CR patients initially demonstrated higher levels of PD-L1 or PD-1 compared to the NR. This part of our data indicates the importance of establishing more sensitive and specific algorithms for clinical use when evaluating AML patients with a low/high risk. We must also differentiate between predictions of a long-term, relapse-free survival following a therapy and a response to the applied chemotherapy. Some studies indicated that higher immune cell-related levels of immune checkpoint proteins were associated with a reduced disease-free time or the general survival of AML patients [39,40,41]. Furthermore, certain analyses focused above all on the assessment of the influence that the level of immune checkpoints in the bone marrow of AML patients had on their survival and not in the peripheral blood, as presented here [14].

Immunosuppressive cytokines, such as IL-10 or TGF-beta, have been to date predominantly considered as unfavourable elements in leukaemia in the presence of their excess. In contrast, pro-inflammatory IL-1beta and IL-6 were expected to demonstrate active anti-tumour activity [42,43]. We were not able to separate the blast- and lymphocyte-related production of the mentioned cytokines; thus, we reported that IL-6 could originate from both populations [44]. On the other hand, cytokines such as IL-17 and IL-10/TGF-beta were rather predominantly related to specific populations—Th17 and Treg, respectively [45]—allowing us to identify their source with high probability. Nonetheless, the current study focused exclusively on the general cytokine environment and its eventual links to the other monitored parameters. In combination with the immune checkpoint data, PD-1+ CD25+ T cells produce more IL-10 than cells without the surface expression of that protein [36]. Novel studies revealed that these cytokines were associated with a blocked expansion of AML blasts. It was also linked to an excessive inflammatory response that, instead of limiting, could promote AML progression [42,43]. We presume that unremarkable changes in cytokines tested here are associated with maintaining a balance between the pro- and anti-inflammatory factors. Thus, the observed increase in IL-1beta and IL-6 in PD-1 or PD-L1 blocking was followed by an elevation of TGF-beta. Cytokines play a crucial role in the course of AML development and progression, predominantly when proper balance is disturbed [42,43]. In accordance, at the last stage of our study, we aimed at evaluating responses of some selected immune proteins to an in vitro therapy of our patients’ blasts and lymphocytes with checkpoint inhibitors. The lack of IL-17A role in AML—mostly produced by Th17 cells—has been reported so far in the context of, inter alia, the general survival. A crucial association of the cytokines with survival was demonstrated in IL-6 as an indirect link and in IL-10 as a direct correlation. A similar significance of those cytokines was reported in our study in reference to the response to the therapy with immune checkpoint inhibitors [43]. We additionally revealed that, in certain tested conditions, even the seemingly irrelevant cytokines, such as TNF-alpha or IFN-gamma, can be associated with the responses of blasts and lymphocytes to the applied therapy.

The essential aspects of evaluating the suitability of certain selected immune checkpoint inhibitors for AML therapy are the related side effects. CLTA-4 inhibitors (ipilimumab) are associated with significantly more adverse reactions when compared to the blockers of PD-1 or PD-L1 (nivolumab/pembrolizumab or atezolizumab, respectively) [46]. Although promising, experimental studies on PD-1/PD-L1 axis inhibition did not provide fully satisfactory results on its possible implementation in de novo AML therapy. Nevertheless, its suitability in a consolidation and maintenance approach or combination with hypomethylating agents (HMAs) still requires verification [23,47]. Novel predictors were described recently for planning an effective use of anti-PD-L1 antibodies. AML patients with high inflammatory responses were more likely to benefit from the blocking of immune checkpoint proteins [42]. That could indicate a possible limitation of our study, as the actual effectiveness of PD-L1 blockers might require an evaluation of the inflammatory potential of the patients. In contrast to the presented negative correlation of CTLA-4, PD-1, or PD-L1 with the long-term survival of AML patients [38,39,40,41], we provided evidence that those data do not exclude the scenario of a beneficial response to the applied chemotherapy. Another limitation of our study is the evaluation of immune checkpoint inhibitors on the newly diagnosed AML patients. Further studies would be essential to compare responses between the initially treated subjects and, at some later stages, subjects with a relapsed/refractory AML. To date, a few studies suggested the beneficial effects of blocking checkpoint proteins in the first group of patients [22,48].

## 5. Conclusions

In conclusion, we demonstrated in vitro some beneficial effects of anti-PD-L1 antibodies on the activation status of T cells, together with a possible maintenance of a proper immune balance through the induction of the Treg phenotype. Despite no substantial efficacy of the tested immune checkpoint blockers on AML blast viability, the modulation of PD-1/PD-L1 axis positively influenced the proliferation of lymphocytes. Patients with a favourable reaction to the subsequent chemotherapy showed a higher expression of PD-1 and PD-L1 and not CLTA-4. Having described the essential phenomenon of inhibiting immune checkpoint proteins, we firmly believe that more attention should be paid to the immune microenvironment associated with the tumour cells. It is worth noting that the tested drugs do not specifically affect the direct actions of the chemotherapeutics, such as cytarabine. Thereby, we should rather expect the anti-cancer immune responses to be enhanced. Antibodies aimed at CTLA-4, PD-1, or PD-L1 could constitute a significant complementation of the current approaches in the management of AML. However, further studies ought to be conducted with a view of establishing the profiles of patients who would benefit more from checkpoint inhibitors and the development of specific drug regimens for more significant clinical results.

## Data Availability

The raw data supporting the conclusions will be made available by the authors upon request.

## Supplementary Materials

The following supporting information can be downloaded at https://www.mdpi.com/article/10.3390/cancers16020462/s1. Figure S1. Patients’ characteristics and methodological background. Description of AML patients enrolled in the study, including comparison of CR and NR groups (A). Presentation of the gating strategy implemented in assessment of blast and lymphocyte viability (B) and proliferation (C). Gating strategy used for evaluation of CD25+ and CD127+ within CD4+ (Th) or CD8+ (Tc) lymphocytes (D) and frequency of regulatory T cells (E). Figure S2. Justification of the research approach implemented in the study. Effects of cytarabine application at the concentration selected for the study on viability of lymphocytes and blast cells after 48 h in vitro incubation (A). Basal ex vivo levels of CTLA-4, PD-1, and PD-L1 within peripheral blood lymphocytes and blast cells of untreated AML patients (B). Presentation of blast cells and lymphocytes viability after 48 h of incubation without stimulation and therapeutics (C). Demonstration of the cytarabine in vitro effects on the proliferation of lymphocytes and blast cells in 96 h cell culture of AML patients’ PBMC (D). Figure S3. Presentation of the flow cytometric controls used in the study. Cells stained with colcemide were used to gate CFSE-stained proliferating CD13+/CD33+ blasts and lymphocytes. CD13+/CD33+ blasts were gated using unstained control (A). Dead 7AAD+ cells were distinguished using unstained control for lymphocyte populations or CD13+/CD33+ single-stained control for blast cells (B). Gating for CD25+ and CD127+ lymphocytes was based on the CD4 or CD8 FMO controls independently (C). For delineation of CD4+ or CD8+ T cells, FMO control was stained with anti-CD8 or anti-CD4, respectively. Similarly, T cells with CD25+CD127- were gated on the basis of CD25 and CD127 FMO controls, considering high expression of CD25 only and negative/low CD127 in CD4+ lymphocytes (D). Figure S4. Demonstration of absolute concentrations of the cytokines analysed in the cell culture supernatants. Data were presented as median values and interquartile ranges. Basal differences in cytokines concentration (IL-1beta, TNF-alpha, IFN-gamma, IL-6, IL-10, TGF-beta, IL-17), between studied groups and treatments, were included additionally. Figure S5. Analysis of correlations between cytokines and immune cell related parameter responses to in vitro therapy. Data demonstrated separately for CR and NR patients from three different layouts, including anti-CTLA-4 (A), anti-PD-1 (B), and anti-PD-L1 (C) antibodies.
