# Supplementary figures and images for: Effects of Combinatory In Vitro Treatment with Immune Checkpoint Inhibitors and Cytarabine on the Anti-Cancer Immune Microenvironment in De Novo AML Patients
Source: Cancers (Basel). 2024 Jan 22;16(2):462. doi: 10.3390/cancers16020462 (PMC10814928; doi:10.3390/cancers16020462)

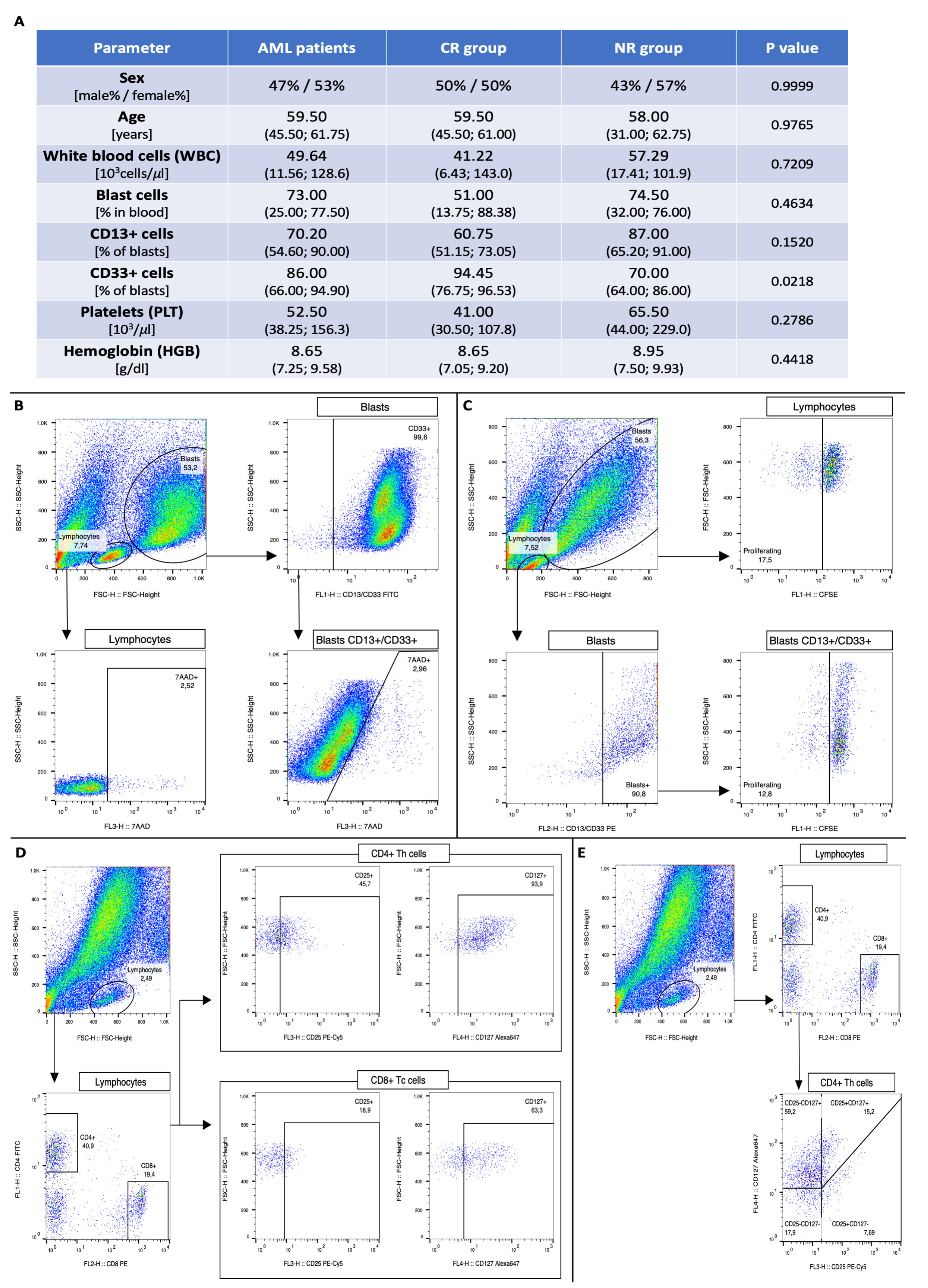

Supplement: Supplementary file 1 [file cancers-16-00462-s001.zip › Supp. Fig.1.jpg]

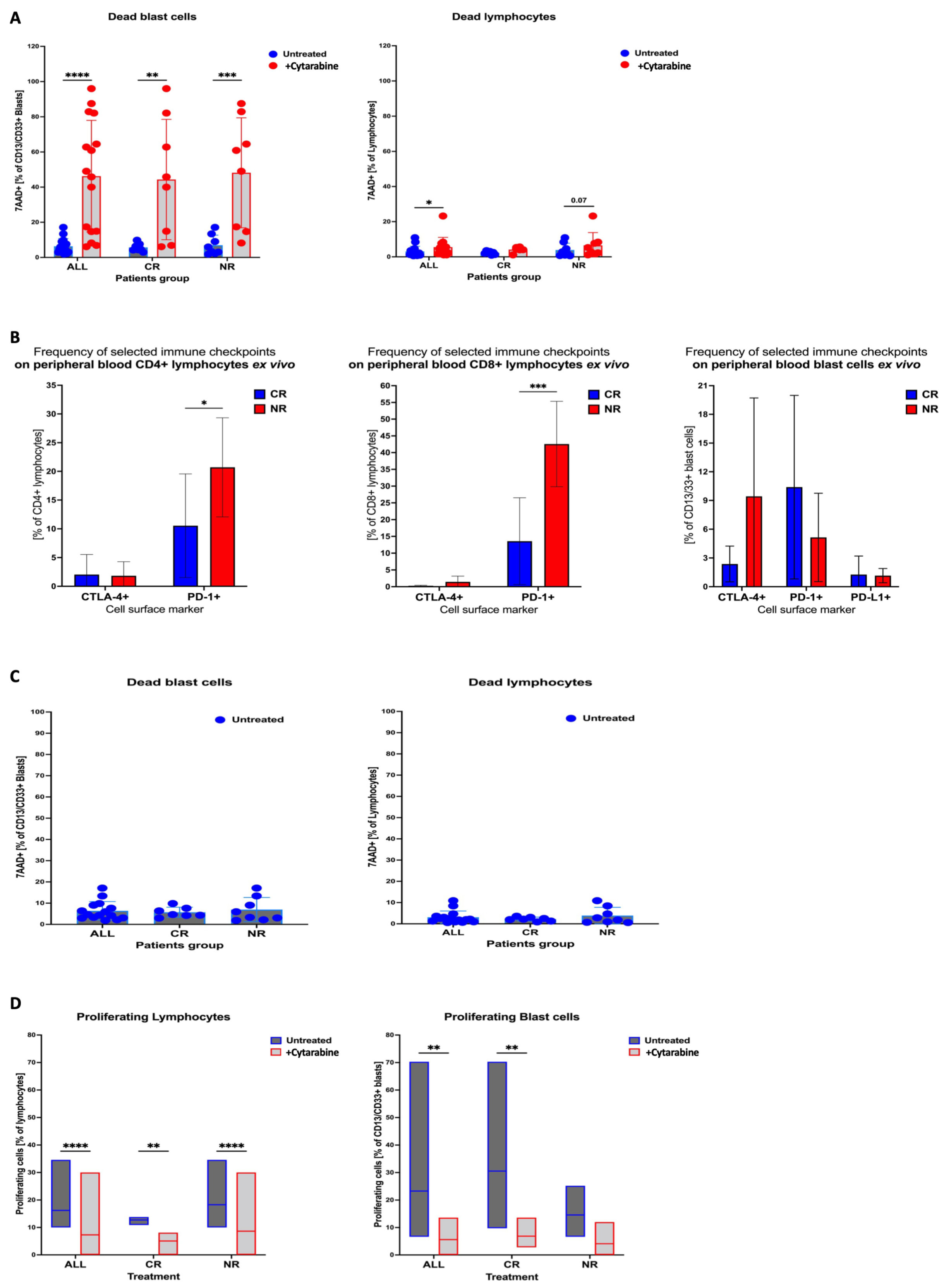

Supplement: Supplementary file 1 [file cancers-16-00462-s001.zip › Supp. Fig.2.jpg]

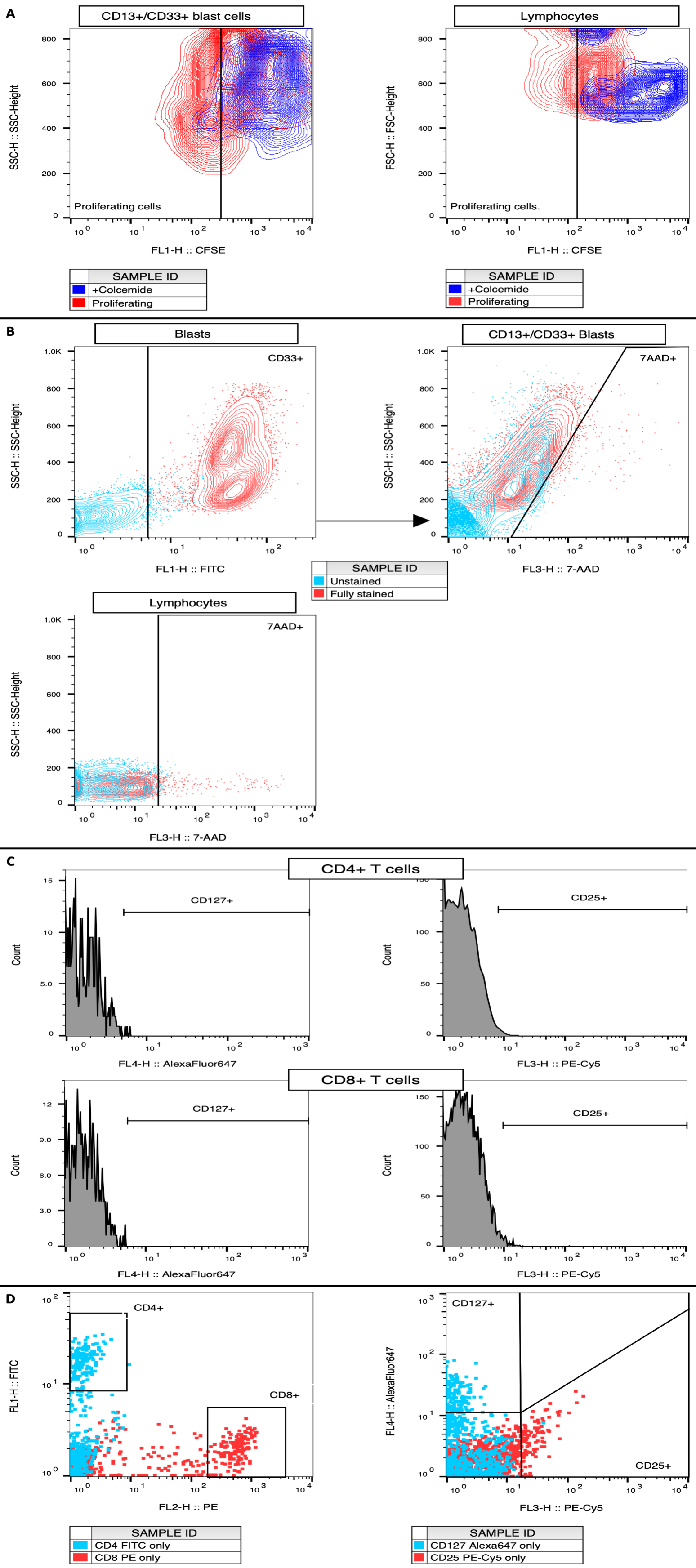

Supplement: Supplementary file 1 [file cancers-16-00462-s001.zip › Supp. Fig.3.jpg]

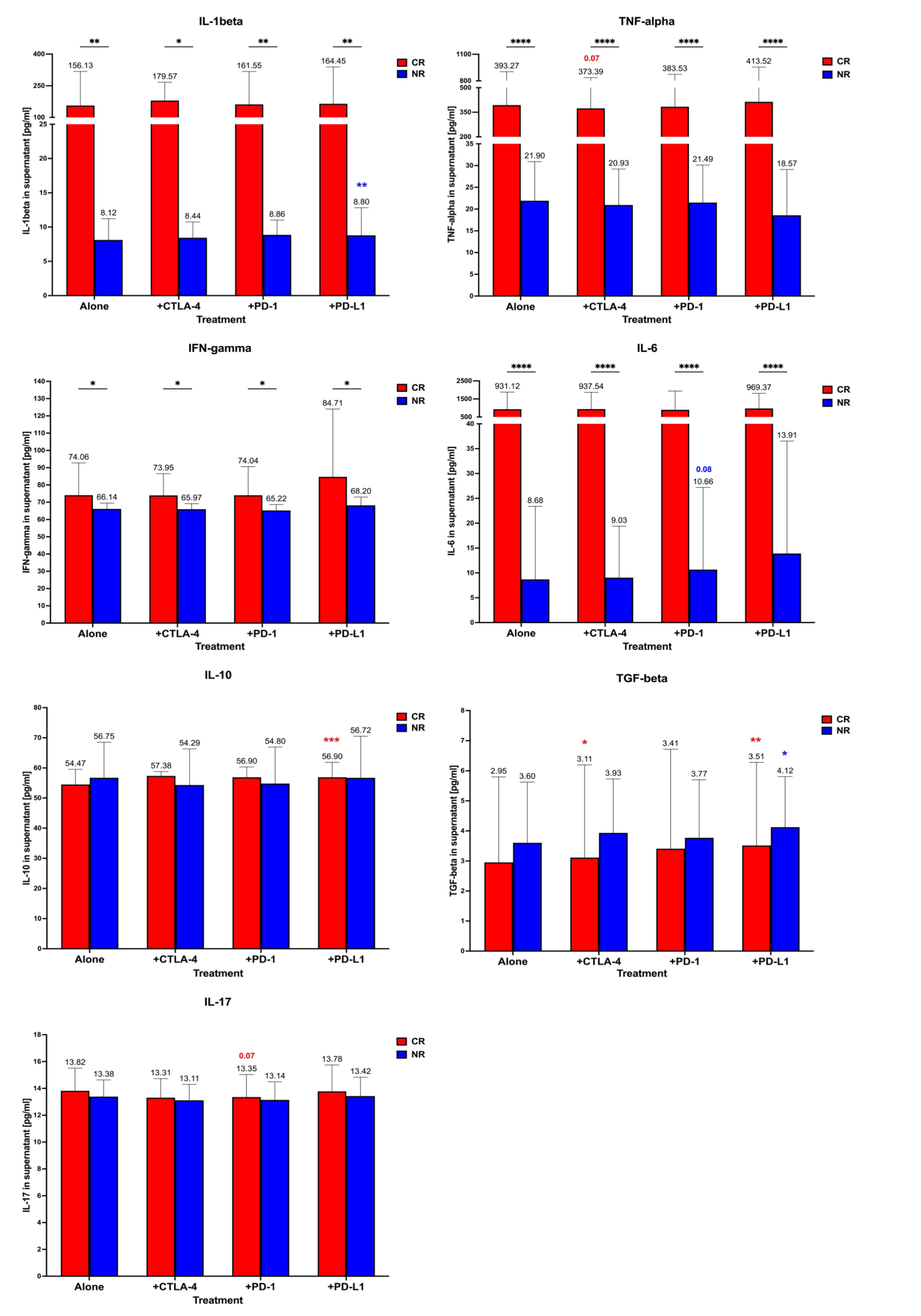

Supplement: Supplementary file 1 [file cancers-16-00462-s001.zip › Supp. Fig.4.jpg]

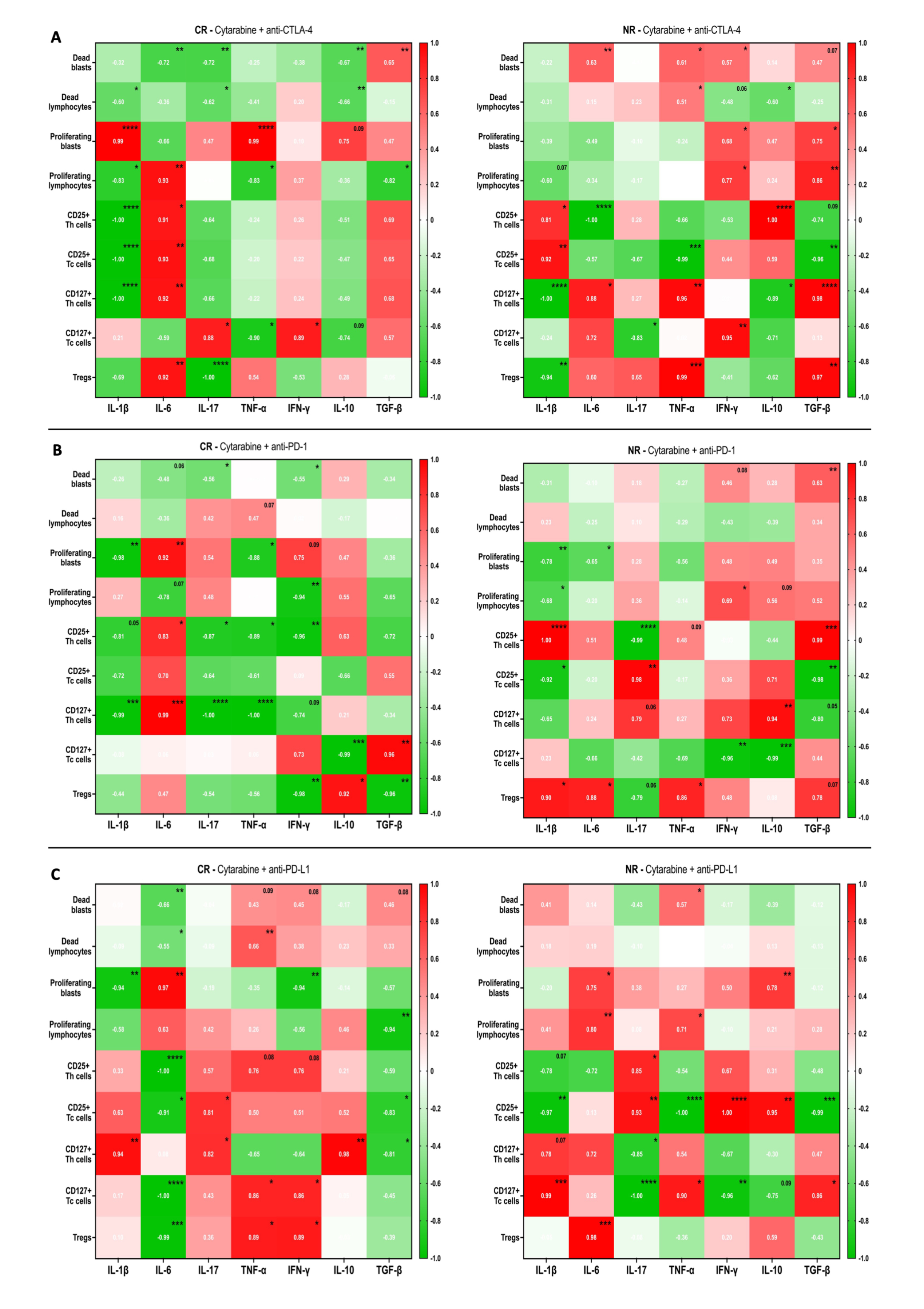

Supplement: Supplementary file 1 [file cancers-16-00462-s001.zip › Supp. Fig.5.jpg]
